# Insulin Protects Hepatic Lipotoxicity by Regulating ER Stress through the PI3K/Akt/p53 Involved Pathway Independently of Autophagy Inhibition

**DOI:** 10.3390/nu8040227

**Published:** 2016-04-19

**Authors:** Hua Ning, Zongxiang Sun, Yunyun Liu, Lei Liu, Liuyi Hao, Yaxin Ye, Rennan Feng, Jie Li, Ying Li, Xia Chu, Songtao Li, Changhao Sun

**Affiliations:** 1 Department of Nutrition and Food Hygiene, Public Health College, Harbin Medical University, Harbin 150086, China; ninghua188@126.com (H.N.); sun1981214@163.com (Z.S.); 13156904466@163.com (Y.L.); leier9211@163.com (L.L.); 18686841061@163.com (L.H.); 15244619691@163.com (Y.Y.); Fengrennan@163.com (R.F.); lijie.hmu@gmail.com (J.L.); liying_helen@163.com (Y.L.); cx831128@163.com (X.C.); 2 Research Institute of Food, Nutrition and Health, Sino-Russian Medical Research Center, Harbin Medical University, Harbin 150086, China; 3 Harbin Center for Disease Control and Prevention, Harbin 150086, China; 4 Key Laboratory of Cardiovascular Medicine Research (Harbin Medical University), Ministry of Education, Harbin 150086, China

**Keywords:** insulin, lipotoxicity, hepatocytes, autophagy, NAFLD

## Abstract

The detrimental role of hepatic lipotoxicity has been well-implicated in the pathogenesis of NAFLD. Previously, we reported that inhibiting autophagy aggravated saturated fatty acid (SFA)-induced hepatotoxicity. Insulin, a physiological inhibitor of autophagy, is commonly increased within NAFLD mainly caused by insulin resistance. We therefore hypothesized that insulin augments the sensitivity of hepatocyte to SFA-induced lipotoxicity. The present study was conducted via employing human and mouse hepatocytes, which were exposed to SFAs, insulin, or their combination. Unexpectedly, our results indicated that insulin protected hepatocytes against SFA-induced lipotoxicity, based on the LDH, MTT, and nuclear morphological measurements, and the detection from cleaved-Parp-1 and -caspase-3 expressions. We subsequently clarified that insulin led to a rapid and short-period inhibition of autophagy, which was gradually recovered after 1 h incubation in hepatocytes, and such extent of inhibition was insufficient to aggravate SFA-induced lipotoxicity. The mechanistic study revealed that insulin-induced alleviation of ER stress contributed to its hepatoprotective role. Pre-treating hepatocytes with insulin significantly stimulated phosphorylated-Akt and reversed SFA-induced up-regulation of p53. Chemical inhibition of p53 by pifithrin-α robustly prevented palmitate-induced cell death. The PI3K/Akt pathway blockade by its special antagonist abolished the protective role of insulin against SFA-induced lipotoxicity and p53 up-regulation. Furthermore, we observed that insulin promoted intracellular TG deposits in hepatocytes in the present of palmitate. However, blocking TG accumulation via genetically silencing DGAT-2 did not prevent insulin-protected lipotoxicity. Our study demonstrated that insulin strongly protected against SFA-induced lipotoxicity in hepatocytes mechanistically through alleviating ER stress via a PI3K/Akt/p53 involved pathway but independently from autophagy.

## 1. Introduction

Nonalcoholic fatty liver disease (NAFLD), which is becoming the most common cause of chronic liver disease, encompasses a spectrum of conditions, ranging from accumulation of lipids in liver (steatosis) to fatty alterations with inflammation and hepatocellular injury or fibrosis (nonalcoholic steatohepatitis, NASH), and even to cirrhosis and liver failure [1]. Evidence has accumulated indicating that NAFLD represents a principal hepatic manifestation of the metabolic syndrome [2]. Although the underlying mechanisms contributing to the pathogenesis of NAFLD are not fully understood, lipotoxicity has been identified as a substantial risk for the metabolic syndrome, including NAFLD [3,4].

Hepatic lipotoxicity referred to the ectopic deposition of excess lipids in the hepatocytes under the circumstance of hyperlipidemia. It is well-accepted that saturated fatty acids (SFAs), such as palmitate and stearate, account for the most parts of lipotoxicity, whereas unsaturated fatty acids (USFAs), such as oleate, generally confer protection against SFA-induced hepatotoxicity [5]. The potential mechanisms involved in SFA-induced lipotoxicity in hepatocytes are not fully illustrated; however, a well-accepted idea is that endoplasmic reticulum (ER) stress and oxidative stress are the major intracellular responses activated by palmitate exposure and contribute to the SFA-induced lipotoxicity via a p53 involved pathway [6,7]. Alleviating SFA-induced hepatotoxicity provides a conceivable strategy for protecting against the development of NAFLD.

In addition to the lipotoxicity, insulin resistance is another hallmark of NAFLD, which is accompanied by reduced insulin sensitivity in the liver and increased circulating insulin level [8]. The detrimental effect of insulin resistance and accompanying increased circulating insulin have been identified in several aspects and in multiple tissues, such as disturbing energy metabolism, promoting hepatic steatosis and TG accumulation in adipose tissue, and also followed by aggravated inflammation, which conform to the pathogenesis of NAFLD [9,10,11].

Autophagy is a reparative life-sustaining process by which cytoplasmic components are sequestered in double-membrane vesicles and degraded upon fusion with lysosomal compartments [12]. Previously, we have reported that the activation of autophagy protected against SFA-induced lipotoxicity in human hepatocytes [13] and high-fat diet (HFD)-induced hepatic steatosis [14]. Similar evidence was also obtained from different experimental settings in various types of cells [15,16]. Moreover, impaired autophagic flux has been observed in the liver from both NAFLD patients and HFD-induced murine models of NAFLD [17], which might play a critical role in NAFLD, as supported by our previous observations that inhibiting autophagy significantly aggravated hepatotoxicity [13,18]. More recently, insulin has been implicated in the inhibition of autophagy via a phosphatidylinositol 3-kinase (PI3K)/Akt-regulated mammalian target of rapamycin (mTOR) pathway [19]. However, the physiological role of insulin-mediated inhibition of autophagy in SFA-induced hepatotoxicity remains largely unknown. Based on the previous reports, we hypothesized that the increased insulin sensitizes SFA-induced hepatotoxicity and mechanistically contributes to the development of NAFLD via regulating autophagy. Unexpectedly, our results demonstrated that insulin prevented SFA-induced lipotoxicity in hepatocytes. The following mechanistic study revealed that the insulin-activated PI3K/Akt pathway is involved in this protection via blocking SFA-induced ER stress and p53, independently of autophagy inhibition and TG accumulation.

## 2. Materials and Methods

### 2.1. Chemicals

Insulin (I3536), palmitic acid (P0500), stearic acid (S4751), oleic acid (O1008), bovine serum albumin (A7030), LY294002 (L9908, inhibitor of PI3Kα/β/δ), and Pifithrin-α (p4236, inhibitor of p53) were purchased from Sigma-Aldrich (St. Louis, MO, USA). Other chemicals used in this study were purchased from different companies, and were shown as follows: rapamycin (LC Laboratories, Woburn, MA, USA, R-5000), bafilomycin A1 (LC Laboratories, Woburn, MA, USA, B-1080, inhibitor of autophagy, functioned via inhibiting the fusion between autophgic and lysosome), chloroquine (Enzo Life Sciences, Farmingdale, NY, USA, 0219391910, inhibitor of autophagy, functioned via inhibiting the activity of lysosome), Earle’s Balanced Salt Solution (HyClone Laboratories, Logan, UT, USA, SH30029.02), MTT Cell Proliferation and Cytotoxicity Assay Kit (C0009), and Hoechst 33258 (C1011) were purchased from beyotime Institute of Biotechnology (Nantong, China), fetal bovine serum (PAA Laboratories, GmbH, Pashing, Austria, A15-701). Palmitic acid-, stearic acid-, and oleic acid-BSA conjugates were prepared as described previously [20]. Briefly, each kind of fatty acid was dissolved in ethanol and saponified with sodium hydroxide. The sodium salt was dried, re-suspended in saline and heated at 80 °C until it completely dissolved. While the solution was still warm, isovolumetric 20% (*w*/*v*) BSA was added and the mixture was stirred at 50 °C for 4 h to allow the acids to bind to BSA. Palmitic acid-, stearic acid-, and oleic acid-BSA complex (3 mmol/L fatty acid: 1.5 mmol/L BSA; molar ratio, 2:1) was then sterilized by filtering for the future usage.

### 2.2. Animal and Experimental Protocol

All protocols in this study were approved by the Medical Ethics Committee of Harbin Medical University (Harbin, China) and were performed in accordance with the National Institutes of Health regulations for the care and use of animals in research. The approval code is 81472981. Sixteen c57bl/6 mice (male, 8-week old) obtained from Beijing Vital River Laboratory Animal Technology Co., Ltd. (Beijing, China) were maintained at a 12:12 h light: dark cycle, and given water ad libitum. Animals were housed in an environmentally controlled room at 21 ± 2 °C, and 50% ± 5% humidity. The mice were randomly divided into normal diet group (ND, *n* = 8, 63.8% carbohydrate, 20.3% protein, and 15.9% fat) and high-fat diet group (HFD, *n* = 8, 40.5% carbohydrate, 17.1% protein, and 42.4% fat) fed for 8 weeks. Plasma and liver tissue were collected for further measurements.

For analyzing insulin’s *in vivo* regulations of autophagy, thirty c57bl/6 mice (male, 8-week old) were fasted overnight (lasting 16 h). Insulin (0.1 U/g BW, Lantus SoloStar, 5B024A) was administered via intraperitoneal injection. After the indicated time points, mice (*n* = 5) were sacrificed and the plasma and liver tissue were collected for further measurements.

### 2.3. Animal Sample Analysis

Plasma free fatty acids (FFAs), were determined using Gas Chromatography-Mass Spectrometer (GC-MS) as described in our previous study [21]. Plasma ALT and insulin were detected using ALT test Kit (Wako, Osaka, Japan, 996-10801) or mouse insulin ELISA Kit (Mercodia, Uppsala, Sweden, 10-1247-01) according to the manufacturer’s instructions, respectively. Triglyceride (TG) deposits in the liver tissue were measured using a Triglyceride test Kit (ApplyGen, Beijing, China, E1013) according to the manufacturer’s instructions.

### 2.4. Cell Culture

Human liver carcinoma cell line (HepG2), obtained from American Type Culture Collection (ATCC, HB8065), was routinely grown in Dulbecco’s Modified Eagle Medium (DMEM, Sigma-Aldrich, St. Louis, MO, USA, D5648) cultural medium, containing 10% (*v*/*v*) fetal bovine serum (PAA Laboratories, GmbH, Pashing, Austria, A15-701), 100 U/ml penicillin, and 100 μg/mL streptomycin (Life Technologies, Gaithersburg, MD, USA, 15140-122). Alpha mouse liver (AML)-12 hepatocyte was established from a mouse transgenic for human transforming growth factor α, and was obtained from the ATCC (CRL-2254), and was cultured in DMEM/Ham’s Nutrient Mixture F-12, 1:1 (DMEM/F-12, Sigma-Aldrich, St. Louis, MO, USA, 051M8322) containing 10% (*v*/*v*) fetal bovine serum, 5 mg/mL insulin (Sigma-Aldrich, St. Louis, MO, USA I3536), 5 μg/mL transferrin (Sigma-Aldrich, St. Louis, MO, USA, T8158), 5 ng/mL selenium (Sigma-Aldrich, 229865), 40 ng/mL dexamethasone (Sigma-Aldrich, St. Louis, MO, USA, D4902), 100 U/mL penicillin, and 100 μg/mL streptomycin. The cultured medium was deprived of insulin when the experiments were performed on AML-12 cells, except for the indicated adding. For all the *in vitro* tests, the insulin level in the control group was zero, and in the experimental groups was described as indicated. All the hepatocytes were cultured at 37 °C in a humidified O_2_/CO_2_ (95:5) atmosphere.

### 2.5. Cell Death Assays

Cell death was determined by the measurements of LDH release, MTT test, and Hoechst staining. In LDH assay, culture medium was collected and detected using LDH assay kit (Thermo Scientific Inc, Waltham, MA, USA, NC9674653) according to the manufacturer's instructions. For MTT test, MTT (0.5 mg/mL) was added after the indicated treatment and incubated at 37 °C for 4 h. MTT test was performed to detect absorbance at 550 nm by microplate reader (M2, MD, CA). In Hoechst staining, cells were stained with Hoechst staining solution (beyotime, C1011) according to the manufacturer’s instructions and imaged by Nikon eclipse Ti-S fluorescence microscope (Nikon, Tokyo, Japan). For propidium iodide staining, cells were trypsinized and stained with propidium iodide staining solution (BD Pharmingen, San Diego, CA, USA) according to the manufacturer’s instructions. Fluorescence was measured using flow cytometry.

### 2.6. ROS Detection

Intracellular ROS levels were detected as previously described [22]. After the indicated treatment, cells were washed with PBS for 3 times, and incubated with 20 µM 2′,7′-dichlorofluorescin diacetate (DCFH-DA, Sigma-Aldrich, D6883) in PBS at 37 °C for 30 min. DCFH fluorescence was measured by Nikon Ti-S fluorescence microscope (Nikon, Tokyo, Japan). The results were normalized to fluorescence intensity of the control group.

### 2.7. Analysis of RFP-LC3 Puncta

The mRFP-GFP-LC3 plasmid was kindly provided by Tom Wileman (University of East Anglia) [23]. Cells were transient transfected with mRFP-GFP-LC3 plasmid using Lipofectamine 2000 (Invitrogen, Carlsbad, CA, USA, 11668) according to the manufacturer’s instructions. Puncta was detected by Nikon eclipse Ti-S fluorescence microscope (Nikon, TYO, Japan). At least 50 cells were counted in each individual experiment.

### 2.8. Western Blot Analysis

Western blot was performed as described previously [24] and the following antibodies were used: anti-Caspase3 (9662), anti-PARP1 (9532), anti-p53 (2524), anti-phospho-Akt (13038), anti-Akt (4691), anti-LC3B (3868), anti-Grp94 (20292), anti-Grp78 (3177), anti-CHOP (5554), and anti-Atg5 (12994) from Cell Signaling Technology Inc (Beverly, MA, USA); β-actin (sc-58679) from Santa Cruz Biotechnology (Santa Cruz, CA, USA).

### 2.9. RNA Interference

Cultured cells were transfected with human Atg5 siRNA (sc-41445) from Santa Cruz Biotechnology (Santa Cruz, CA, USA) using Lipofectamine 2000 according to the manufacturer’s instructions. In the control group, cells were transfected with scrambled siRNA (Santa Cruz Biotechnology, Santa Cruz, CA, USA, sc-37007).

### 2.10. Statistical Analysis

All data were expressed as mean ± S.D. except for special instruction. The statistical analyses were performed with the SPSS V20.0 (IBM Corp, Armonk, NY, USA) program using *t*-test or one-way ANOVA, followed by the Student-Newman-Keuls test. All *p*-values were two-tailed, and a *p*-value < 0.05 was considered significant for all statistical analyses.

## 3. Results

### Subsection

#### SFAs Induce Hepatotoxicity in Human Hepatocytes

**Figure 1:**
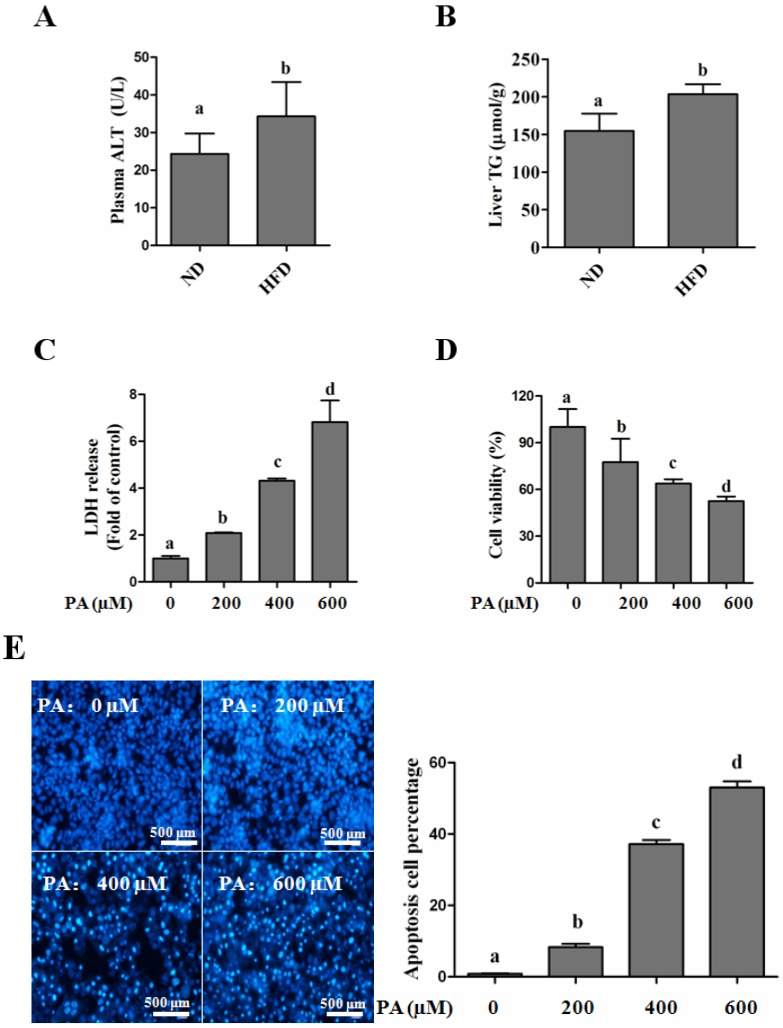

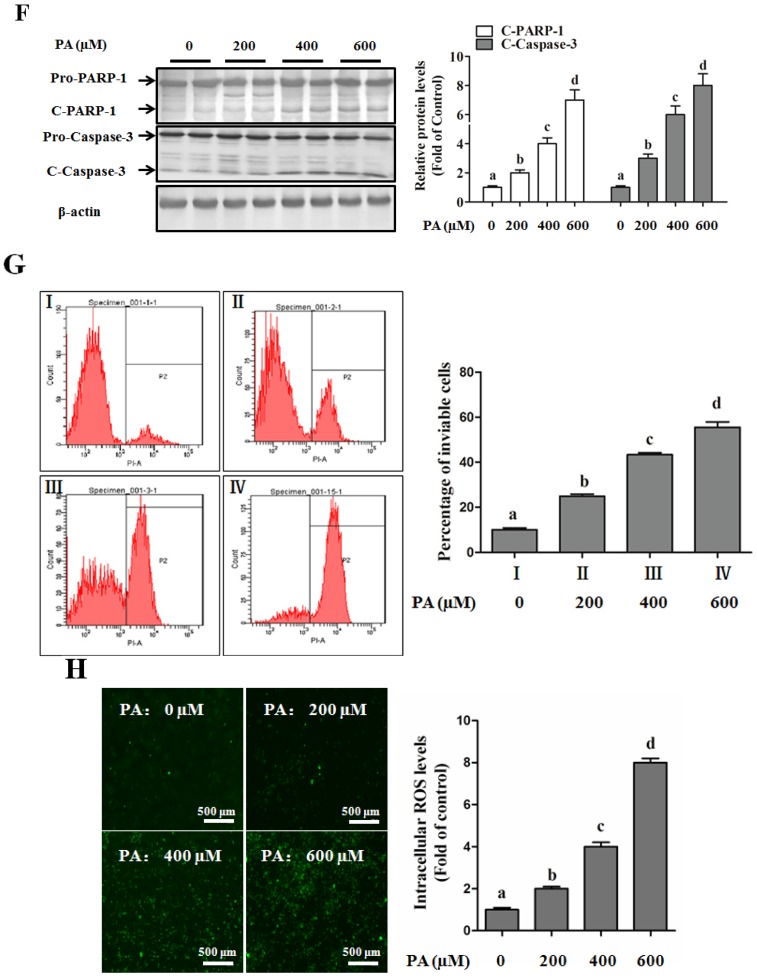
SFA-induced hepatic lipotoxity in c57bl/6 mice and HepG2 cells. C57bl/6 mice were fed with a normal diet (ND) or high-fat diet (HFD) for 8 weeks. Plasma ALT and hepatic TG contents were detected as described in the Materials and Methods. HepG2 cells were treated with palmitic acid (PA) with the indicated dosage for 12 h. (**A**) Plasma ALT (*n* = 8); (**B**) liver TG (*n* = 8); (**C**) LDH in the cultured medium was detected as described in the Methods; (**D**) Cell viability was detected by MTT test; (**E**) Nuclear morphology was detected by Hoechst staining using fluorescence microscopy (magnification × 100); (**F**) Total lysates of HepG2 cells were subjected to immunoblotting assay for Caspase-3 and PARP-1; (**G**) Cell death was dectected by propidium iodide staining with flow cytometry; (**H**) Intracellular ROS production was measured by DCFH-DA stain using fluorescence microscopy (magnification × 100). Each *in vitro* test was performed at least 3 times. Bars with different characters differ significantly (*p* < 0.05).

HFD, a more clinically relevant diet, was employed in this study to induce NAFLD according to the previous studies [25]. After 8 weeks of HFD feeding, more severe injury was observed in the HFD group, evidenced by the detection of plasma ALT (Figure 1A). The TG accumulation in the liver tissue was significantly higher in the HFD group than that in the ND mice (Figure 1B). Additionally, the FFAs profile was robustly altered by HFD feeding, among which total FFA and SFA levels were significantly increased (Table 1). Subsequently, we tested SFA-induced hepatotoxicity in HepG2 cells. In line with previous studies, our results revealed that palmitate exhibited significantly lipotoxicity in a dose-dependent manner in human hepatocytes, which was supported by the LDH, MTT, and PI staining (Figure 1C,D,G), Besides, the nuclear morphological measurement was performed via staining the nuclei with Hoechst 33258. We observed that the nuclei in the control group maintain integrity, while PA (400 µM and 600 µM) significantly increased the number of condensed nuclei, which were more brightly stained. (Figure 1E). The intracellular protein indicators for damaged cells, including cleaved-parp-1 and -caspase-3, were markedly up-regulated by palmitate incubation (Figure 1F). Besides, palmitate treatment also enhanced intracellular ROS production (Figure 1H). The similar hepatotoxicity was also observed when replacing palmitate with stearic acid, an 18-carbon chain saturated fatty acid (Figure S1).

#### Autophagy Protects against Palmitate-Induced Lipotoxicity

**Figure 2:**
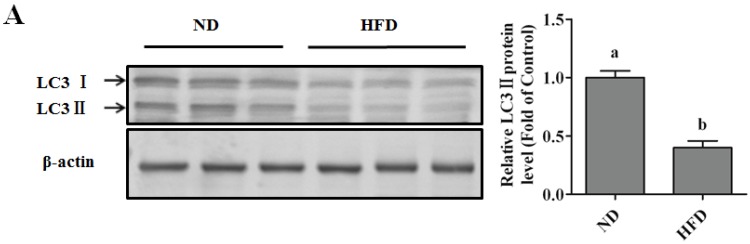

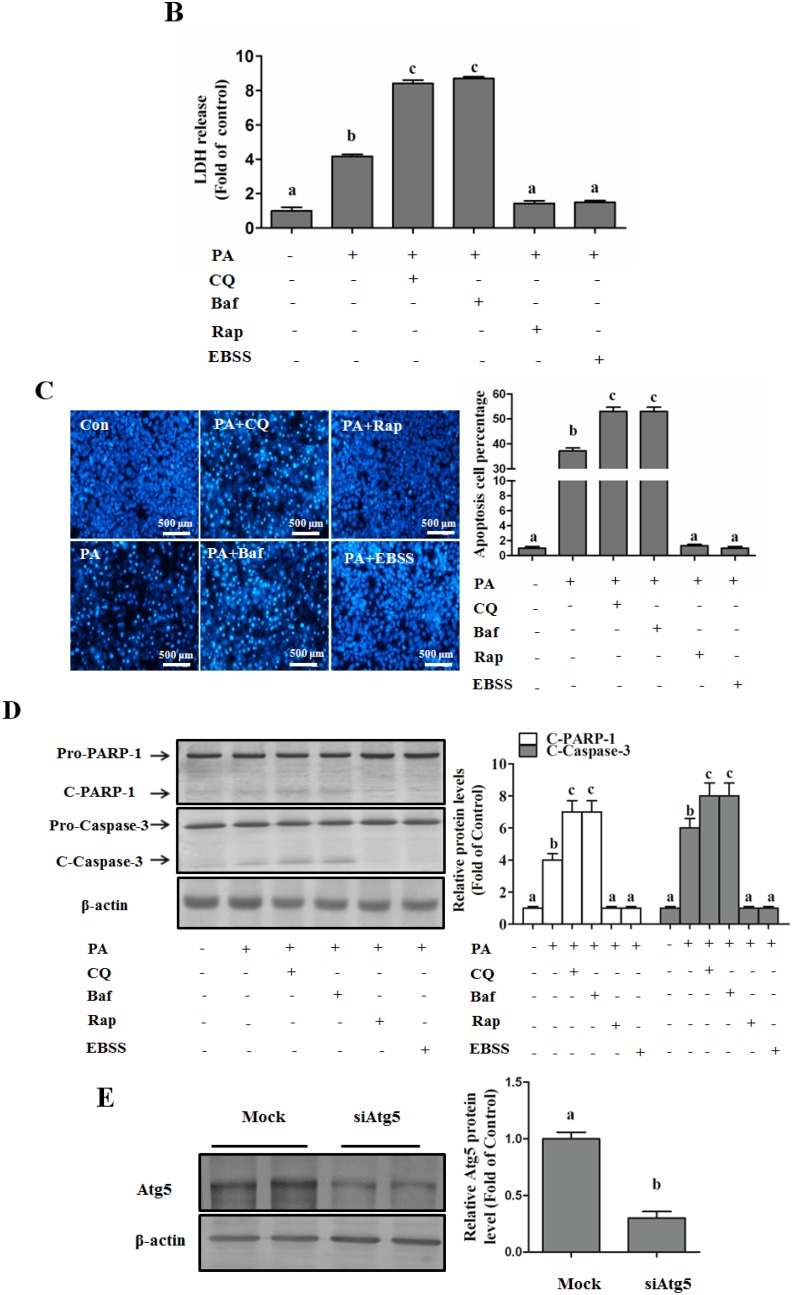

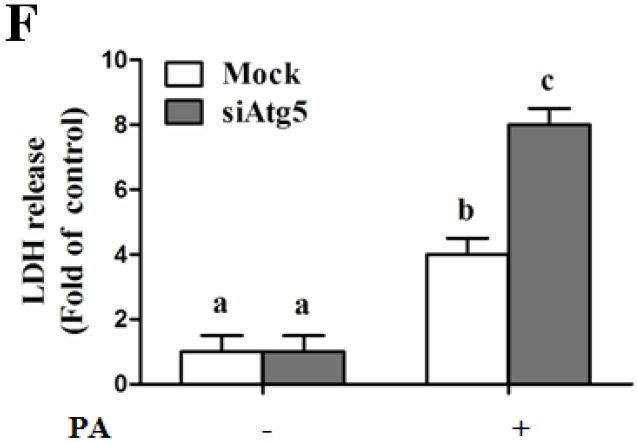
Regulating autophagy contributes to SFA-induced lipotoxicity in hepatocytes. HepG2 cells were treated with palmitic acid (PA, 400 µM) for 12 h with or without rapamycin (Rap, 1 μM), Earle’s Balanced Salt Solution (EBSS), chloroquine (CQ, 20 μM), or Bafilomycin A1 (Baf, 100 nM) pre-treatment for 4 h. (**A**) Liver protein was extracted using RIPA lysis. Immunoblotting was performed for LC3; (**B**) LDH in the cultured medium was detected as described in the Methods; (**C**) Nuclear morphology was detected by Hoechst staining using fluorescence microscopy (magnification × 100); (**D**) Total lysates of HepG2 cells were subjected to immunoblotting assay for Caspase-3 and PARP-1; (**E**) Cells were transfected with either scrambled or Atg5 siRNA. Transfected efficiency was detected by immunoblotting assay; (**F**) After transfected with Atg5, cells were treated with palmitic acid (PA, 400 µM) for 12 h. LDH in the cultured medium was detected as described in the Methods section. Each *in vitro* test was performed at least 3 times. Bars with different characters differ significantly (*p* < 0.05).

Activation of autophagy protected against SFA-induced cytotoxicity in various types of cells, such as rat pancreatic beta-cells, mouse hepatocytes and human endothelial cells [15,16,18]. Here, we firstly examined the status of autophagy in the liver tissue of HFD-induced NAFLD mice, we observed that the autophagy was more significantly inhibited in the HFD group than that in the normal diet group, evidenced by the lower LC3-II formation existing in the NAFLD mice liver (Figure 2A). The effect of autophagy inducers on palmitate-induced cell death in HepG2 cells was subsequently detected. Cells were pretreated with the well-accepted autophagy inducer rapamycin or cultured with EBSS medium to mimic starvation before palmitate exposure. We observed that autophagy played a protective role in palmitate-induced cell death in HepG2 cells, which was determined by LDH, nuclear morphous, and cleaved-parp-1 and -caspase-3 expressions measurements (Figure 2B–D). Conversely, pretreating cells with special inhibitors of autophagy, CQ or bafilomycin, obviously exacerbated palmitate-induced hepatotoxicity (Figure 2B–D). Genetically inhibiting autophagy using siRNA targeted Atg5 also enhanced palmitate-induced cell death (Figure 2E,F).

#### Autophagy Protects against Palmitate-Induced Lipotoxicity

**Figure 3:**
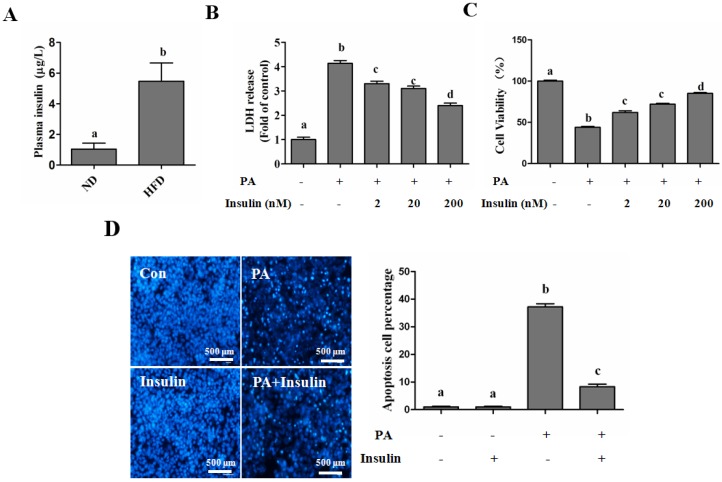

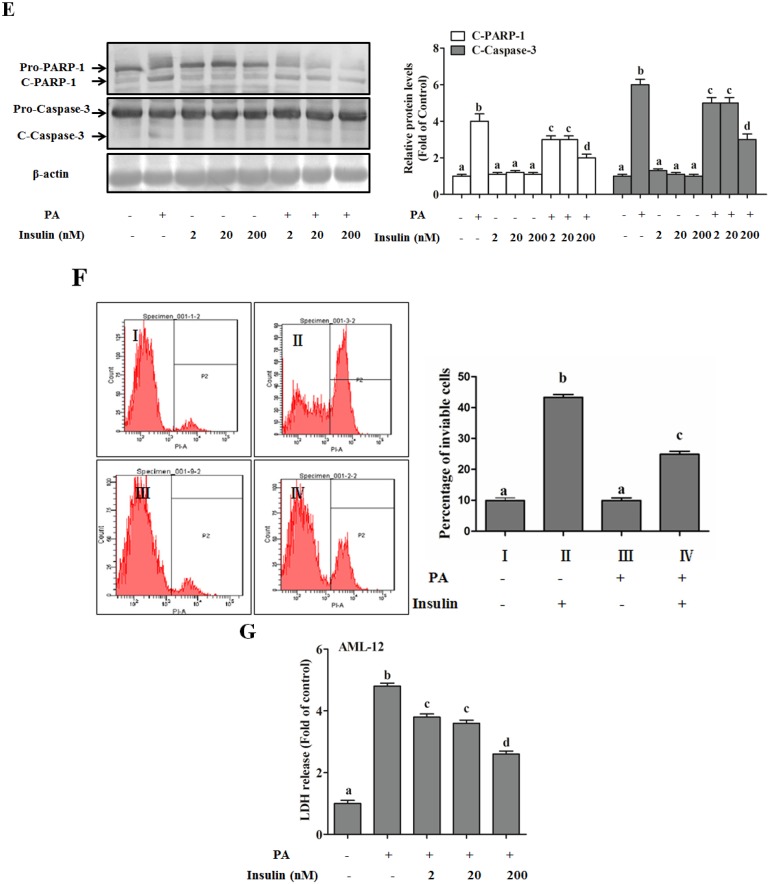
Insulin protects against SFA-induced hepatotoxicity. Mouse plasma insulin level was detected as described in the Materials and methods (*n* = 8). HepG2 cells were exposed to palmitic acid (PA, 400 µM) with or without different dose of insulin pretreatment for 1 h. (**A**) Plasma insulin; (**B**) LDH in the cultured medium was detected as described in the Methods; (**C**) Cell viability was detected by MTT test; (**D**) Nuclear morphology was detected by Hoechst staining using fluorescence microscopy (magnification × 100); (**E**) Total lysates of HepG2 cells were subjected to immunoblotting assay for Caspase-3 and PARP-1; (**F**) Cell death was dectected by propidium iodide staining with flow cytometry; (**G**) AML-12 hepatocytes were used to replace HepG2 cells. LDH in the cultured medium was detected as described in the Methods. Each *in vitro* test was performed at least 3 times. Bars with different characters differ significantly (*p* < 0.05).

In this study, plasma insulin level was significantly elevated in HFD-induced NAFLD mice (Figure 3A). Pretreating cells with a progressive dose of insulin presented a markedly protective role against palmitate-induced cell death (Figure 3B,C,E,F). In nuclear morphological measurements (Figure 3D), the nuclei in the control group and insulin group maintained integrity and 400 µM PA treatment significantly increased the number of condensed nuclei, while insulin decreased the number of PA-damaged nuclei. The lipotoxicity was also alleviated by insulin when replacing pamitate by stearate (Figure S2). Moreover, in a non-transformed mouse hepatocyte cell line (AML-12), palmitate-induced cell death was significantly blocked by insulin treatment (Figure 3G).

#### Autophagy Is Independent from Insulin-Protected Lipotoxicity in Hepatocytes

**Figure 4:**
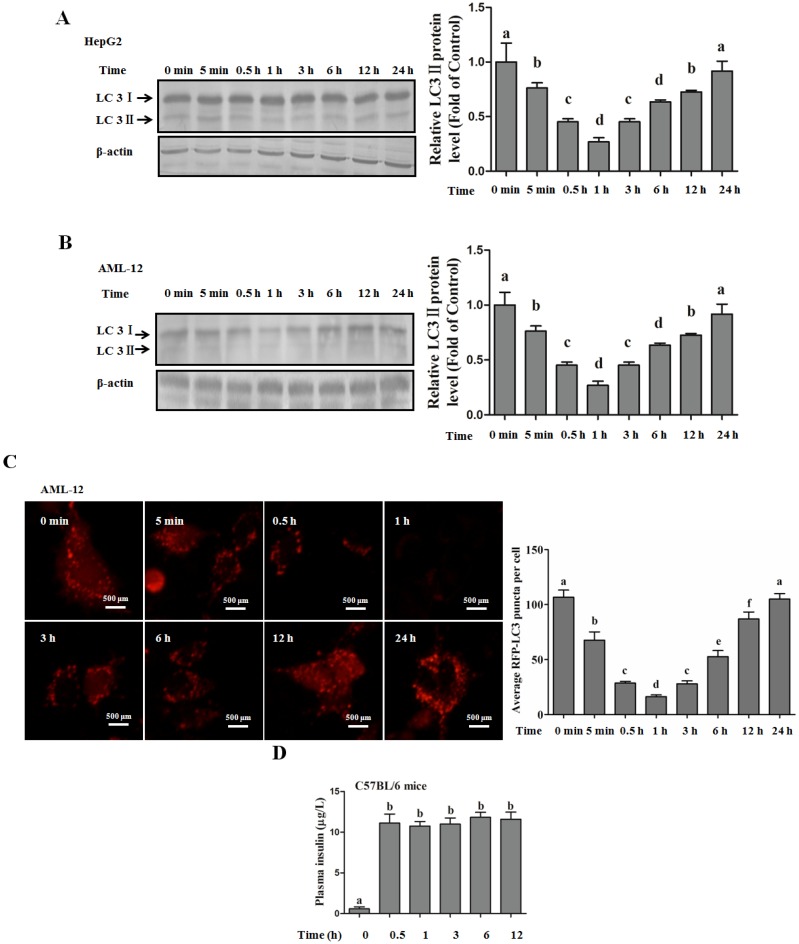

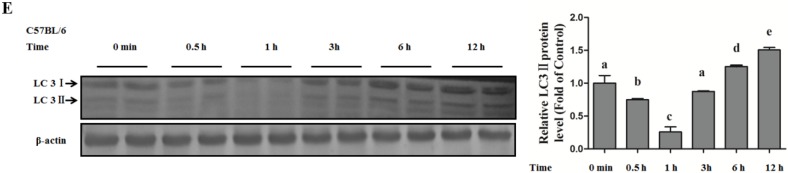
Time course-dependent regulation of insulin on hepatic autophagy. Hepatocytes were cultured within EBSS medium for 12 h, and then exposed to insulin (200 nM) for the indicated duration. Total lysates of cells were subjected to immunoblotting assay for LC3. Plasmid (containing mRFP-GFP-LC3) transfection was performed 12 h before EBSS intervention for autophagosome detection. For *in vivo* analysis of hepatic autophagy, mice were suffered to insulin injection as described in the Methods. (**A**,**B**) Immunoblotting assay for LC3 conversion in HepG2 cells and AML-12 cells; (**C**) The effect of insulin on autophagy was examined by fluorescence microscopy for puncta formation; (**D**) Mice plasma insulin level (*n* = 5); (**E**) LC3 expression in mice liver (*n* = 5). Each *in vitro* test was performed at least 3 times. Bars with different characters differ significantly (*p* < 0.05).

**Figure 5:**
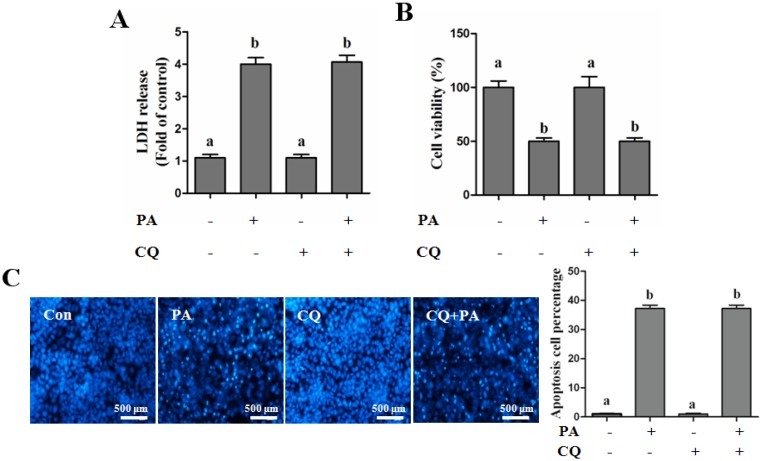
Short-term inhibition of autophagy does not aggravate palmitate-induced hepatic lipotoxicity. HepG2 cells were exposed to autophagy inhibitor (CQ, 20 μM) for 1 h, after then the cultural medium was totally replaced by the fresh medium containing only palmitic acid (PA, 400 µM) and treated for 12 h. The lipotoxicity was detected. (**A**) LDH in the cultured medium was detected as described in the Methods section; (**B**) Cell viability was detected by MTT test; (**C**) Nuclear morphology was detected by Hoechst staining using fluorescence microscopy (magnification × 100). Each test was performed at least 3 times. Bars with different characters differ significantly (*p* < 0.05).

Insulin has been implicated in negative regulation of autophagy via a PI3K/Akt/mTOR-dependent pathway [20]. To further illustrate the reason why insulin inhibits autophagy but does not accelerate palmitate-induced hepatotoxicity, we firstly detected insulin-regulated autophagy in hepatocytes, and our results indicated that insulin-suppressed autophagy could only be observed in the early stage, but was gradually recovered to the normal level after 1 h incubation in both human and mouse hepatocytes, evidenced by the detection of LC3-II (Figure 4A,B). When pre-cultured with EBSS medium, insulin-regulated autophagosome formation was in line with LC3-II expression, which was quantitated by the intracellular fluorescent puncta (Figure 4C). Next, we analyzed insulin’s *in vivo* regulation of hepatic autophagy. The plasma insulin level was significantly increased after intraperitoneal injection (Figure 4D). The hepatic autophagy was rapidly inhibited by insulin, but was gradually recovered after 12 h, which was evidenced by the observation of LC3-II formation (Figure 4E). We also imitated insulin-inhibited autophagy though incubating cells with CQ only for 1 h, and then replenished the cultural medium containing only palmitate, and the result indicated that short-term inhibition of autophagy did not exacerbate palmitate-induced cell death in human hepatocytes (Figure 5A,B). In nuclear morphological measurements (Figure 5C), the nuclei in the control and CQ group maintained integrity and 400 µM PA treatment significantly increased the number of condensed nuclei, while short-term (1 h) CQ intervention presented no additional effect on PA-damaged nuclei.

#### PI3K/Akt-Regulated p53 Pathway Contributes to Insulin-Protected Lipotoxicity

**Figure 6:**
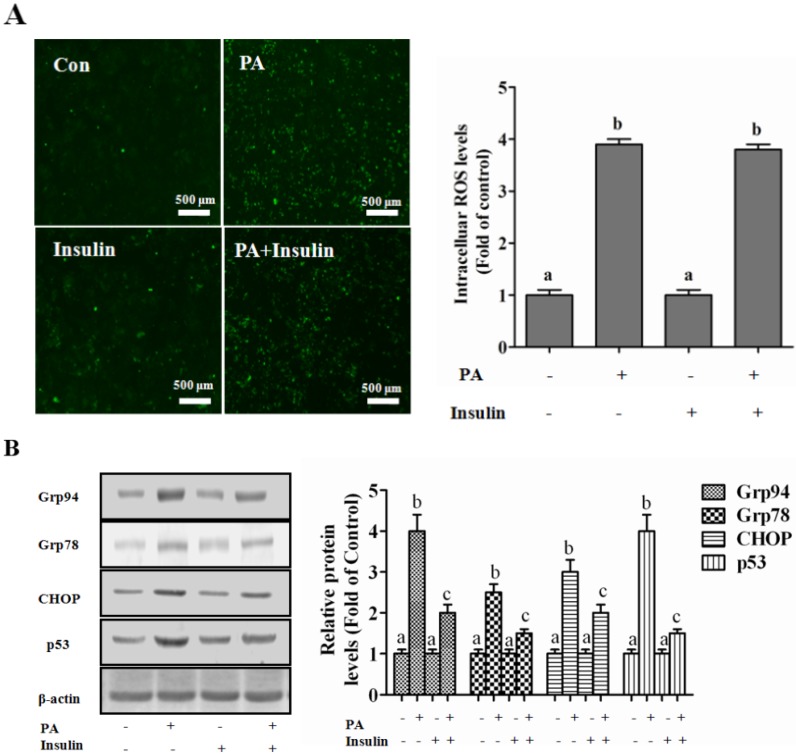

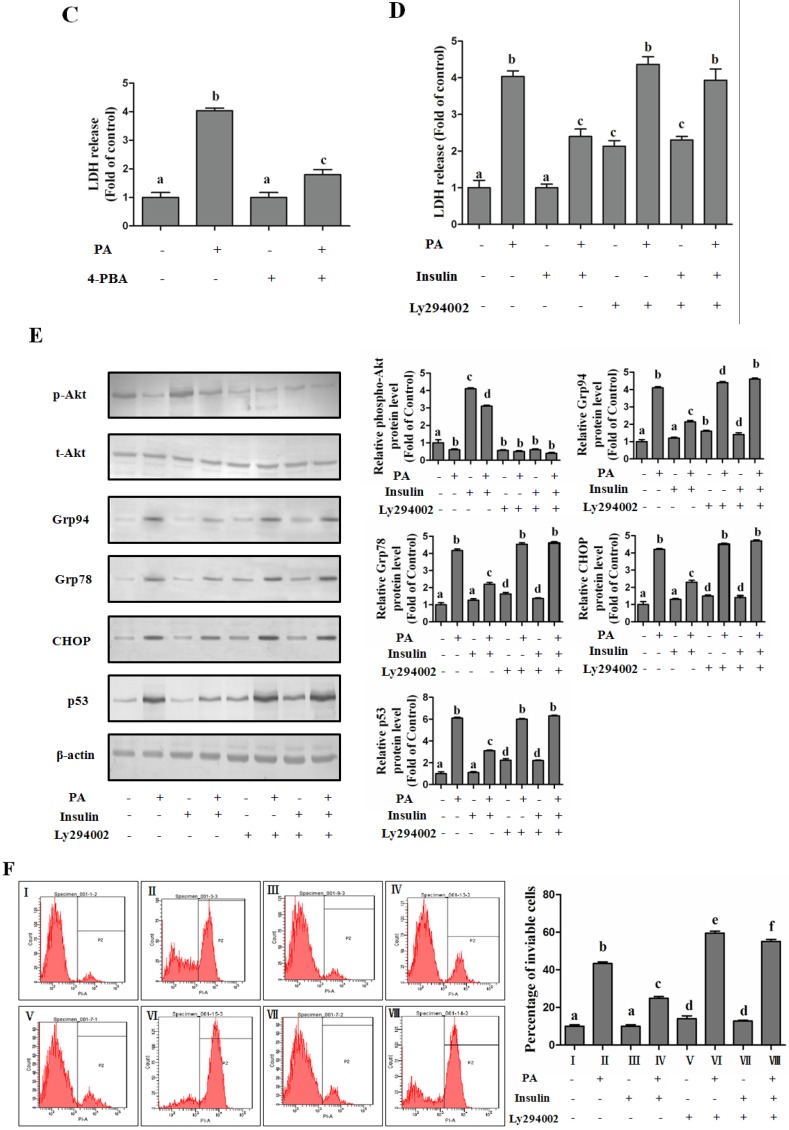

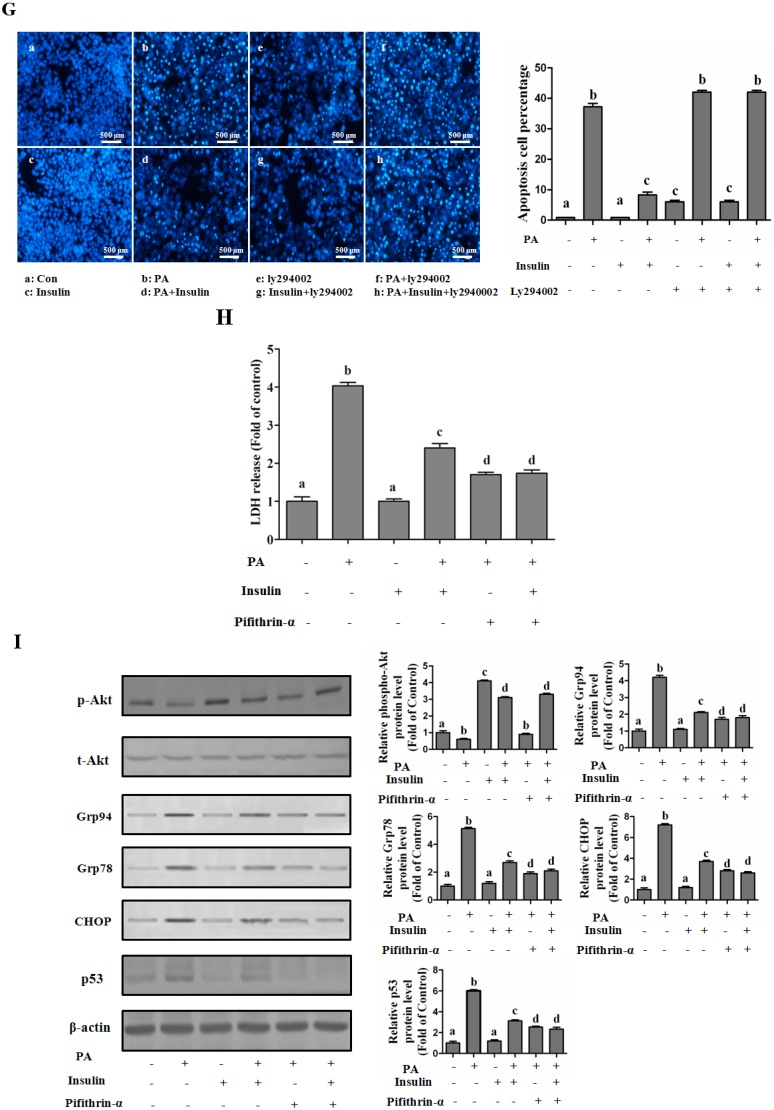

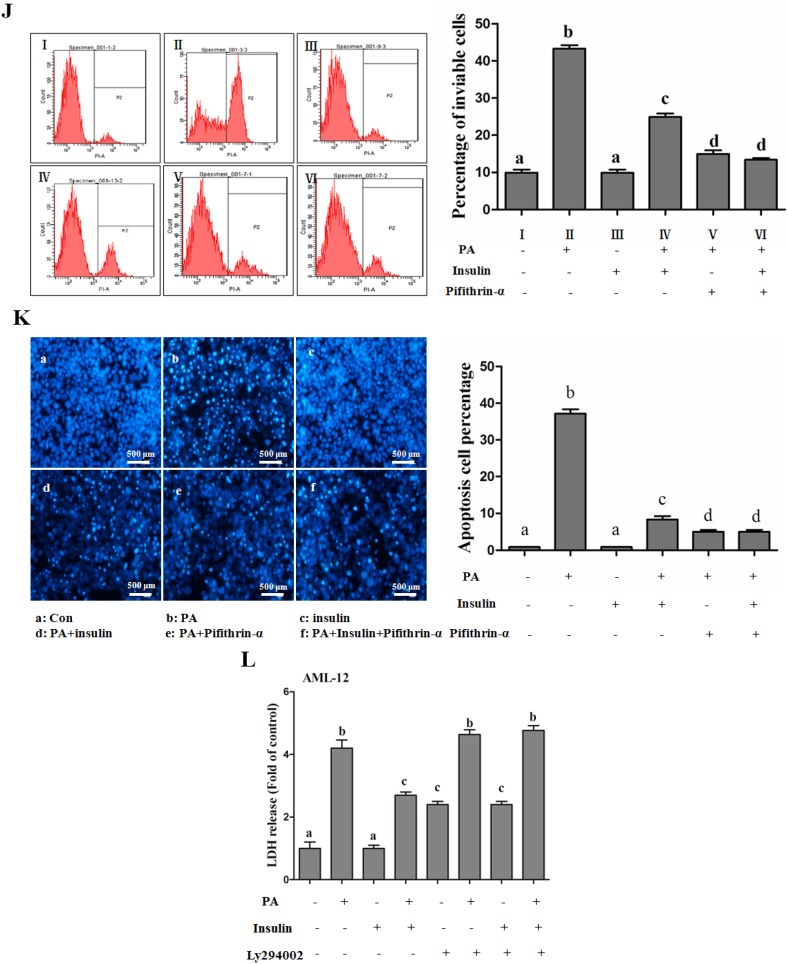
PI3K/Akt-regulated p53 contributes to insulin-protected SFA-induced lipotoxicity via mediating ER stress. HepG2 cells were exposed to palmitic acid (PA, 400 µM) for 12 h with or without insulin (200 nM) pretreatment for 1 h. PI3K/Akt antagonist ly294002 (10 μM) or p53 inhibitor pifithrin-alpha (10 μM) were added 1 h before insulin treatment. The cellular index was detected as follows: (**A**) Intracellular ROS production was measured by DCFH-DA stain using fluorescence microscopy (magnification × 100); (**B**) Immunoblotting assay for GrpP94, Grp78, CHOP, and p53; (**C**) ER stress inhibitor was pretreated for 1 h before exposed to palmitic aced (PA, 400 µM), LDH in the cultured medium was detected as described in the Methods; (**D**,**H**) LDH in the cultured medium was detected as described in the Methods; (**E**,**I**) Immunoblotting assay for phosphorylated Akt, Grp94, Grp78, CHOP p53; (**F**,**J**) cell death was dectected by propidium iodide staining with flow cytometry; (**G**,**K**) Nuclear morphology was detected by Hoechst staining using fluorescence microscopy (magnification × 100); (**L**) AML-12 hepatocytes were used to replace HepG2 cells. LDH in the cultured medium was detected as described in the Methods section. Each test was performed at least 3 times. Bars with different characters differ significantly (*p* < 0.05).

Oxidative stress and ER stress are the well-recognized intracellular pathways stimulated by SFA exposure and contribute to SFA-induced cell injury [26,27]. We firstly assessed the role of insulin on palmitate-driven oxidative stress, and the result indicated that insulin did not reduce palmitate-induced increase in intracellular ROS contents, which rules out the anti-oxidative probability of insulin in the protective processing (Figure 6A). Subsequently, the molecular markers of ER stress, including Grp94, Grp78, and ChOP, were analyzed, and we clearly observed that palmitate-induced ER stress was robustly reversed by insulin exposure (Figure 6B). A well-known pro-apoptosis protein, p53, mediated by ER stress [28], was detected in this study. Our result revealed that palmitate exposure strongly enhanced p53 expression, nevertheless, insulin incubation prevented palmitate-induced p53 up-regulation (Figure 6B). To further investigate the involvement of ER stress in SFA-induced lipotoxicity in hepatocytes, 4-PBA, a well-known ER stress inhibitor, was employed. We observed that 4-PBA significantly ameliorated PA-induced lipotoxicity in hepatocytes (Figure 6C). Moreover, palmitate-suppressed Akt phosphorylation was also apparently reversed by insulin treatment (Figure 6E) chemically inhibiting the PI3K/Akt pathway by its special antagonist. The inhibitory effect of insulin on PA-induced ER stress and cell death was abolished by Ly294002 (Figure 6D–F). Besides, Ly294002 treatment also inhibited the protection of insulin on PA-induced nuclei damage (Figure 6G). Additionally, blocking p53 by pifithrin, a special chemical antagonist, significantly inhibited palmitate-induced ER stress and cell death (Figure 6H,J,K). Pifithrin incubation also alleviated PA-induced increase of condensed nuclei (Figure 6K). Additionally, Ly294002 enhanced palmitate-induced cell death in mouse hepatocytes (Figure 6L). The similar observation was also obtained when replacing palmitate by stearic acids (Figure S3). These results clearly revealed that the PI3K/Akt-regulated p53 pathway was involved in insulin-protected lipotoxicity in hepatocytes.

#### Promoting TG Synthesis Is Absent from the Hepatoprotective Role of Insulin

In contrast to SFA-induced cytotoxicity, unsaturated fatty acids (USFAs) exhibited no effect on cell viability, due to the facility in the synthesis of inert triglyceride (TG) [29]. In this study, we observed that oleic acids, the most abundant USFAs in human circulating system, enhanced TG synthesis and presented no cytotoxicity on HepG2 cells (Figure S4A,B). Moreover, cells incubated with oleic acids robustly abrogated palmitate-induced cell death with an increased intracellular TG accumulation (Figure S4A–D). Insulin has been well-regarded as a physiological factor in spurring anabolism and uptake of TG by adipocytes [30]. In human hepatocytes, we observed that insulin apparently elevated intracellular TG storage when cells suffered palmitate treatment (Figure S5A,B). Palmitate treatment significantly reduced the mature SREBP-1c in the nucleus, but insulin strongly reversed the decrease of SREBP-1c (Figure S6A). We further probed the participation of SREBP-1c in the protective role of insulin. Our results showed that knocking-down SREBP-1c did not block insulin-protected hepatotoxicity or promote intracellular TG accumulation (Figure S6B–D). These results ruled out the participation of SREBP-1c in the protective role of insulin. To further identify the role of increased TG storage in insulin-prevented lipotoxicity, diglyceride acyltransferase (DGAT)-2, the essential enzyme catalyzing the formation of TG from diacylglycerol and acyl-CoA in hepatocytes was investigated. The data revealed that insulin exposure strongly elevated DGAT-2 expression (Figure S7A). Genetically silencing DGAT-2 using its special siRNA significantly blocked insulin-promoted intracellular accumulation of TG, but did not alter the protective role of insulin (Figure S7B–E). These results clearly demonstrated that insulin-induced TG accumulation did not contribute to its hepatoprotective role against lipotoxicity.

## 4. Discussion

The present study aims at clarifying the sensitivity of insulin in SFA-mediated hepatotoxicity in the complicated pathological procedure of NAFLD. We conclude that insulin exhibits a strongly protective role against SFA-induced lipotoxicity in both human and mouse hepatocytes. The results from this study reveal for the first time that insulin-inhibited autophagy is independent from the preventive role, and the further investigations demonstrate that the PI3K/Akt-regulated p53 pathway mechanistically contributes to insulin-protected lipotoxicity in hepatocytes via alleviating ER stress.

Hepatotoxicity induced by SFAs, which has been well-considered as a critical manifestation, plays a pivotal role in the origination and pathological development of NAFLD [3]. In addition to lipotoxicity itself, several endogenous alterations have been observed to sensitize SFA-induced liver injury, such as hyperglycemia and inflammatory cytokines, which probably accelerates the progression of NAFLD [25,26]. Among these alterations, increased circulating glucose-induced toxicity has been largely researched and is implicated in the amplifying of SFAs-led lipotoxicity in hepatocytes [26,27]. Additionally, recent studies have also reported that the genetic changes in NAFLD, including down-regulated phosphorylated-adenosine 5′-monophosphate (AMP)-activated protein kinase (AMPK) and peroxisome proliferator-activated receptors α (PPARα) markedly aggravate SFA-induced hepatotoxicity [18,28]. Therefore, clarifying the sensitivity of lipotoxicity is an urgent task and further provides an ideal therapeutic choice for the treatment of NAFLD.

Autophagy, a highly evolutionarily conserved process, refers to the removal and breakdown of intracellular components (organelles and proteins) via sequestering and targeting bulk components for lysosomal degradation [12]. Emerging evidence suggests that a tightly regulated adaptive mechanism is involved in the autophagy that enhances cell survival under various environmental and cellular stresses though eliminating damaged proteins and organelles [29]. The activation of autophagy exhibits multiple benefits and protects cells against detrimental aggressions [30,31]. Recently, we reported that activating autophagy prevented hepatocytes from palmitate-induced damage in mitochondrial and further apoptosis [13]. In line with this observation, such protection was also existed in palmitate-induced cytotoxicity in pancreas beta-cells and endothelial cells [15,16]. Conversely, inhibiting autophagy aggravated SFA-induced cell injury [32]. Insulin has been reported as playing a negative role in the regulation of autophagy [19]. In the patients with NAFLD or HFD-induced animals, the circulating fasting insulin is commonly elevated due to the insulin resistance [33]. Moreover, insulin injection is a clinically-approved, effectual and widely received way to regulate blood glucose in diabetes and/or NAFLD patients [34,35]. Therefore, we are anxious to know whether increased insulin sensitizes SFA-induced hepatotoxicity via the negative regulation of autophagy and further aggravates the pathological procedure of NAFLD. To answer this question, we firstly confirmed the regulation of autophagy on SFA-induced lipotoxicity in human hepatocytes. Next, hepatocytes were exposed to cultural conditions with both insulin and SFAs. Unexpectedly, pretreating cells with insulin did not enhance the cytotoxicity induced by SFAs; conversely, insulin presented a strongly protective role against the lipotoxicity. It is an interesting task to determine why. The insulin-stimulated PI3K/Akt/mTOR pathway has been implicated in the inhibition of autophagy in some types of tissues [36,37]. However, limited study has addressed the regulative role of insulin on hepatic autophagy, and the conclusions were inconsistent [38,39]. In this study, we investigated the time-course relationship between insulin and hepatic autophagy, and observed that insulin exposure only led to a rapid and short-period inhibition of autophagy, which was gradually recovered after long-term incubation in both cultured hepatocytes and mice liver. Subsequently, we certified that short-term inhibition of autophagy (using special autophagy inhibitors, CQ or Baf) was insufficient to aggravate SFA-induced lipotoxicity, which further explained the reason why insulin exposure did not enhance lipotoxicity. Therefore, autophagy-independent pathways were logically involved in the protective role of insulin.

Subsequently, we investigated the potential mechanisms whereby insulin protected against SFA-induced lipotoxicity. Oxidative stress and ER stress are classical intracellular pathways involved in SFA-induced liver injury. Therefore, we first assessed the anti-oxidative stress role of insulin in palmitate-induced hepatotoxicity. The results indicated that insulin did not reverse palmitate-induced increase of intracellular ROS content, which ruled out the involvement of anti-oxidative mechanism. Another pro-apoptosis pathway induced by palmitate, ER stress, was subsequently considered in this study. A previous study has reported that insulin protects diabetic rats from obesity-induced hepatic ER stress [40]. However, there existed some discrepancy in different experimental settings, in that insulin induced ER stress in adipose tissue and immunocytes [41,42]. In this study, insulin significantly reduced palmitate-stimulated ER stress, evidenced by the down-regulation of the molecular markers, including GRP94, GRP78, and Chop, which clearly indicated that a potential anti-ER stress mechanism contributed to insulin-protected lipotoxicity. Further, the detailed investigations using complementary approaches demonstrated that insulin-activated PI3K/Akt pathway was mechanistically involved in the protection via decreasing palmitate-up-regulated p53. Although the linkage between ER stress and the p53 pathway was not well-investigated in this study, insulin-inhibited stress-activated protein kinases (SAPK) phosphorylation might play a central role in the ER stress-activated p53 pathway in hepatocytes [43].

Unsaturated fatty acids (USFAs), which are prone to construct TG, present no cytotoxicity in hepatocytes and protect against SFA-induced hepatoxicity [44]. In line with previous observations, our study showed that oleic acid, an 18-carbon chain mono-USFA, apparently facilitated the conversion of plamitate into TG, and rescued palmitate-induced hepatotoxicity. Stearoyl-CoA desaturase-1 (SCD-1) is the enzyme that manipulates the switch from SFAs to monounsaturated fatty acids (MUFAs) [45]. *In vivo*, SCD-1^-/-^ mice feeding with a methionine-choline-deficient diet manifested decreased steatosis but markedly increased hepatocellular apoptosis and liver injury when compared to the SCD-1^+/+^ counterparts [46]. The evidence strongly supports the viewpoint that conversing reactive SFAs into inert TG exhibits reduced lipotoxicity in mammalian cells. In this study, we observed that insulin exposure significantly elevated intracellular TG deposits. Therefore, we asked whether insulin-promoted TG accumulation was involved in its protective role. The pro-lipid synthetic pathways were investigated mainly by SREBP-1c and DGAT-2 in this study. Interestingly, genetically silencing either SREBP-1c or DGAT-2 using their siRNA did not impair the protective role of insulin, indicating insulin-promoted TG accumulation was not involved in the protection of SFA-induced hepatoxicity. Although the reason why blocking TG deposits did not affect the protective role of insulin was not illustrated in this study, we presumed that insulin-induced TG accumulation probably originated from glucose conversion, since almost the same extent intracellular TG deposit was induced between only by insulin and by insulin together with palmitate. Additionally, we should not ignore insulin supplementation-triggered TG accumulation in hepatocytes. It is better to consider the conjunctive application of a lipid-reduced drug for the clinical use of insulin in diabetes patients with NAFLD.

## 5. Conclusions

In summary, our study provides strong evidence that insulin exposure protects hepatocytes against lipotoxicity induced by SFAs via a PI3K/Akt/p53 involved pathway through alleviating ER stress. Our results also identify that insulin only inhibits a rapid and short-term autophagy in hepatocytes, which is recovered after long-term exposure, and the protective role of insulin is independent from autophagy.

## Figures and Tables

**Table 1:** Composition of Plasma FFAs in ND and HFD mice.

Free Fatty Acids	ND (μmol/L)	HFD (μmol/L)
C14:0, MA (Myristic acid)	6.76 ± 0.32	5.07 ± 0.08
C16:0, PA (Palmitic acid)	580.88 ± 0.88	730.11 ± 20.11 *
C16:1, PLA (Palmitoleic acid)	73.14 ± 3.95	49.30 ± 1.39 *
C18:0, SA (Stearic acid)	211.88 ± 4.16	383.58 ± 13.59 **
C18:1, OA (Oleic acid)	185.58 ± 8.47	461.37 ± 13.67 **
C18:2, LA (Linoleic acid)	716.22 ± 14.16	919.71 ± 28.19 *
γ-C18:3, γ-LNA (γ-Linolenic acid)	23.18 ± 0.75	22.46 ± 0.81
C18:3, LNA (Linoleic acid)	7.60 ± 0.29	5.54 ± 0.18 *
C20:2, EDA (Eicosadienoic acid)	5.04 ± 0.18	8.08 ± 0.26
C20:4, AA (Arachidonic acid)	383.16 ± 9.88	748.75 ± 24.72 **
C20:5, EPA (Eicosapentaenoic acid)	15.18 ± 0.75	17.87 ± 0.75
C22:5, DPA (Docosapentaenoic acid)	17.48 ± 0.37	23.93 ± 0.47 **
C22:6, DHA (Docosahexaenoic acid)	576.08 ± 12.48	957.23 ± 26.60 **
C24:0, TA (Tetracosanoic acid)	15.24 ± 1.27	17.32 ± 0.81
C24:1, SOA (Selacholeic acid)	17.16 ± 0.47	18.02 ± 0.67
Total fatty acids	2834.59 ± 58.42	4368.35 ± 132.21 **
Saturated fatty acid	814.79 ± 6.61	1136.77 ± 34.44 **
Monounsaturated fatty acids	275.86 ± 12.91	528.68 ± 15.72
Polyunsaturated fatty acids	1743.92 ± 38.84	2703.56 ± 81.97 **
*n*-3 fatty acids	616.32 ± 13.87	1004.56 ± 28.01 **
*n*-6 fatty acids	1127.61 ± 24.95	1698.99 ±53.97 **

ND, normal diet; HFD, high fat diet; Values are means ± SEM; *n* = 8 mice for each group; * *p* < 0.05; ** *p* < 0.01.

## Supplementary Materials

The following are available online at http://www.mdpi.com/2072-6643/8/4/227/s1.

## Abbreviations

The following abbreviations are used in this manuscript:

AML	Alpha mouse liver
AMPK	AMP-activated protein kinase
Baf	Bafilomycin A1
CQ	chloroquine
ER	Endoplasmic reticulum
EBSS	Earle’s Balanced Salt Solution
FFAs	Free fatty acids
HFD	High-fat diet group
HepG2	Human liver carcinoma cell line
MUFAs	Monounsaturated fatty acids
mTOR	Mammalian target of rapamycin
NAFLD	Nonalcoholic fatty liver disease
NASH	Nonalcoholic steatohepatitis
ND	Normal diet group
PI3K	Phosphatidylinositol 3-kinase
PPARα	Peroxisome proliferator-activated receptors α
Rap	rapamycin
SFAs	Saturated fatty acids
SCD-1	Stearoyl-CoA desaturase-1
TG	Triglyceride
USFAs	Unsaturated fatty acids
