# Supplementary material for: Insulin Protects Hepatic Lipotoxicity by Regulating ER Stress through the PI3K/Akt/p53 Involved Pathway Independently of Autophagy Inhibition
Source: Nutrients. 2016 Apr 19;8(4):227. doi: 10.3390/nu8040227 (PMC4848695; doi:10.3390/nu8040227)
Supplement: Supplementary file 1 [file nutrients-08-00227-s001.docx]

Supplementary Materials: Insulin Protects Hepatic Lipotoxicity via Regulating ER Stress through PI3K/Akt/p53 Involved Pathway Independently of Autophagy Inhibition

Hua Ning, Zongxiang Sun, Yunyun Liu, Lei Liu, Liuyi Hao, Yaxin Ye, Rennan Feng, Jie Li,
Ying Li, Xia Chu, Songtao Li and Changhao Sun

1. Supplementary Methods

1.1. Cell Triglyceride Test

Intracellular TG content was performed using either Triglyceride test Kit (ApplyGen, Beijing, China, E1013) or BODIPY®493/503 (Thermo Fisher, D-3922) staining, and detected by microplate reader (M2, MD, CA) or Nikon eclipse Ti-S fluorescence microscope (Nikon, Tokyo, Japan) according to the manufacturer’s instructions, respectively.

1.2. RNA Interference

Cultured cells were transfected with human SREBP-1c siRNA (sc-36557) or si-DGAT2 (sc-44520) from Santa Cruz Biotechnology using Lipofectamine 2000 according to the manufacturer’s instructions. In the control group, cells were transfected with scrambled siRNA (Santa Cruz Biotechnology, sc-37007).

1.3. Western-Blot Analysis

Western-blot was performed as described previously [[25](#_ENREF_25)] and the following antibodies were used: anti-DGAT2 (sc-66859) and anti-histone H3 (sc-8654) from Santa Cruz Biotechnology; anti-SREBP-1c (ab57999) from Abcam.

2. Supplementary Data


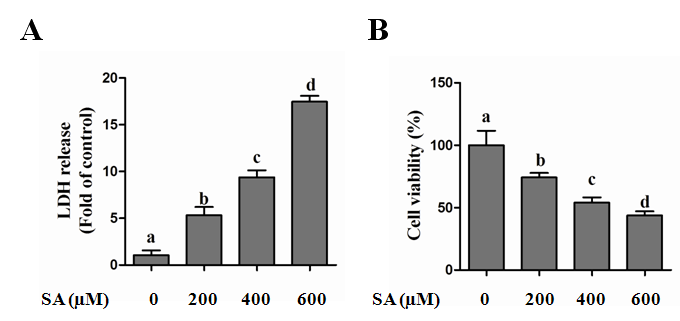


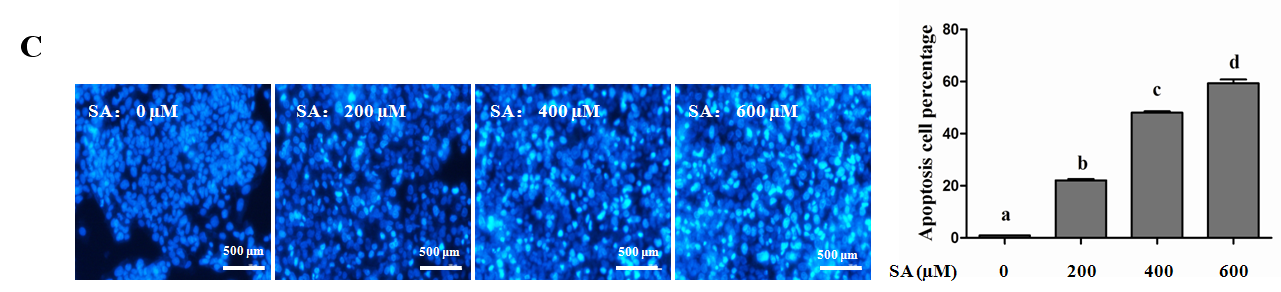


**Figure S1.** Stearate-induced hepatic lipotoxity. HepG2 cells were treated with stearic acid (SA) with the indicated dosage for 12 h. (**A**) LDH in the cultured medium was detected as described in the Methods; (**B**) Cells viability was detected by MTT test; (**C**) Nuclear morphology was detected by Hoechst stainning using fluorescence microscopy (magnification × 100). Each in vitro test was performed at least 3 times. Bars with different characters differ significantly (*p* < 0.05).


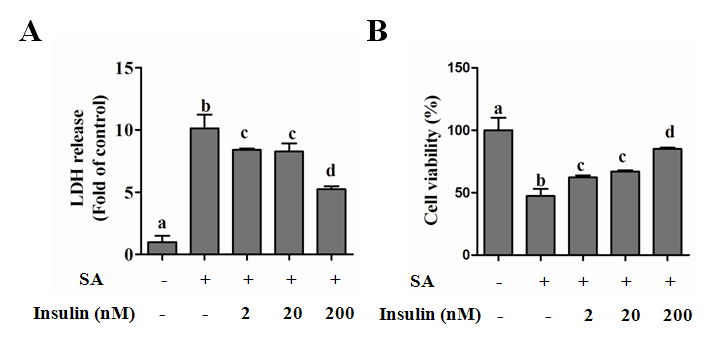


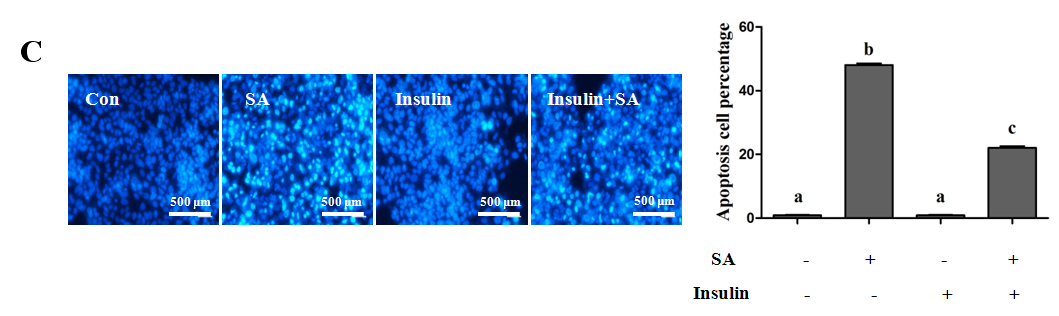


**Figure S2.** Insulin protects against Stearate-induced hepatotoxicity. HepG2 cells were exposed to stearic acid (SA, 400 µM) with or without different dose of insulin pretreatment for 1 h. (**A**) LDH in the cultured medium was detected as described in the Methods; (**B**) Cells viability was detected by MTT test; (**C**) Nuclear morphology was detected by Hoechst stainning using fluorescence microscopy (magnification × 100). Each in vitro test was performed at least 3 times. Bars with different characters differ significantly (*p* < 0.05).


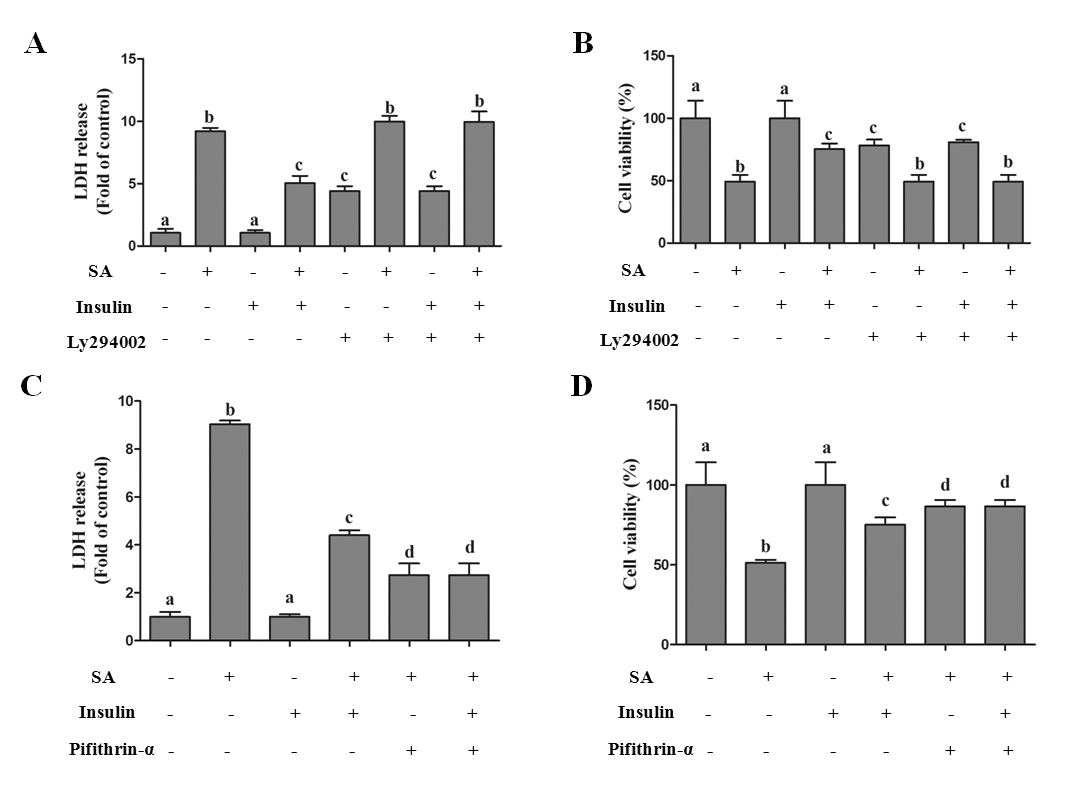


**Figure S3.** PI3K/Akt-regulated p53 contributes to insulin-protected Stearate-induced lipotoxicity. HepG2 cells were exposed to stearic acid (SA, 400 µM) for 12 h with or without insulin (200 nM) pretreatment for 1 h. PI3K/Akt antagonist ly294002 (10 μM) or p53 inhibitor pifithrin-alpha (10 μM) were added 1 h before insulin treatment. (**A**,**C**) LDH in the cultured medium was detected as described in the Methods; (**B**,**D**) Cells viability was detected by MTT test. Each test was performed at least 3 times. Bars with different characters differ significantly (*p* < 0.05).


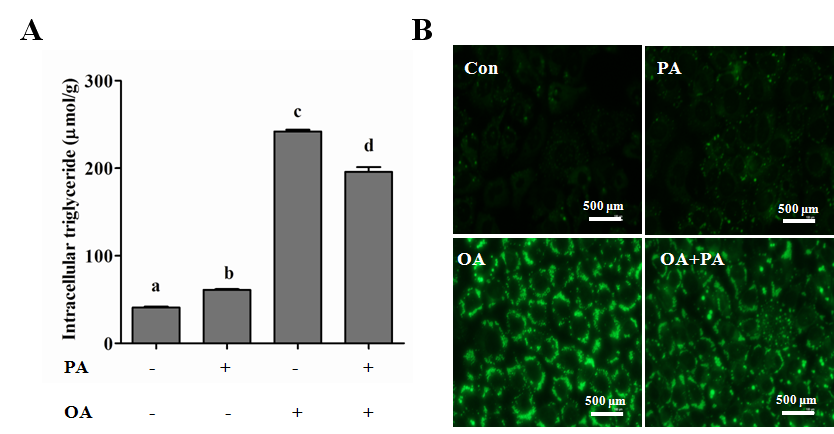


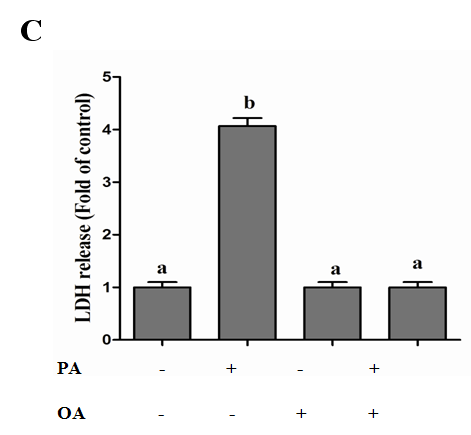


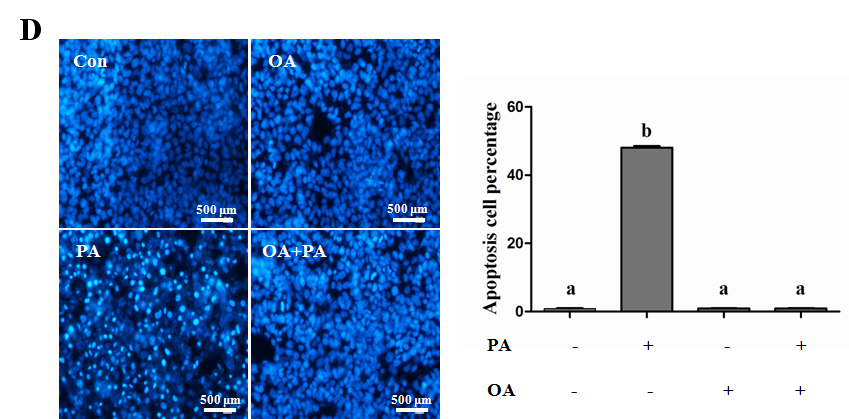


**Figure S4.** Oleic acid enhanced palmitate-induced TG accumulation and inhibited palmitate-induced cell death. HepG2 cells were treated with palmitic acid (PA, 400 µM), oleic acid (OA, 400 µM), or PA (200 µM) and OA (200 µM) combination for 12 h. (**A**) Intracellular triglyceride contents were detected as described in the supplementary methods; (**B**) Intracellular triglyceride accumulation was detected by BODIPY stainning using fluorescence microscopy (magnification × 100); (**C**) LDH in the cultured medium was detected as described in the Methods; (**D**) Nuclear morphology was detected by Hoechst stainning using fluorescence microscopy (magnification × 100). Each test was performed at least 3 times. Bars with different characters differ significantly (*p* < 0.05).


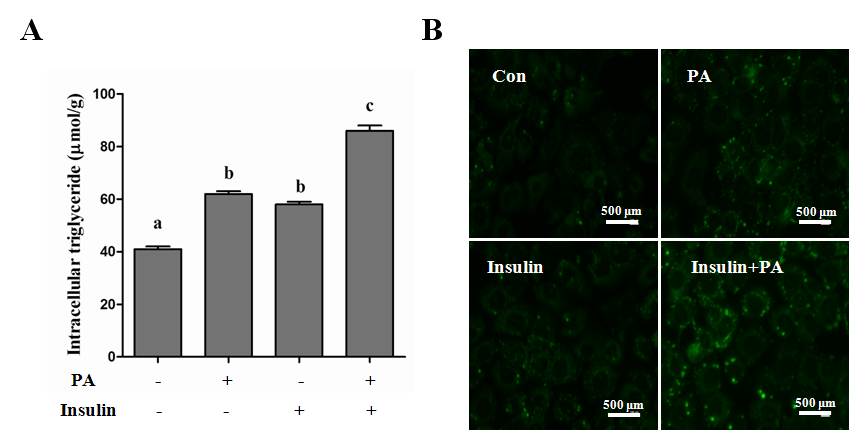


**Figure S5.** Insulin elevated palmitate-induced TG accumulation in HepG2 cells. HepG2 cells were exposed to palmitic acid (PA, 400 µM) with or without insulin (200 nM) pretreatment for 1 h. (**A**) Intracellular triglyceride contents were detected as described in the Materials and methods; (**B**) Intracellular triglyceride accumulation was detected by BODIPY stainning using fluorescence microscopy (magnification × 100). Each test was performed at least 3 times. Bars with different characters differ significantly (*p* < 0.05).


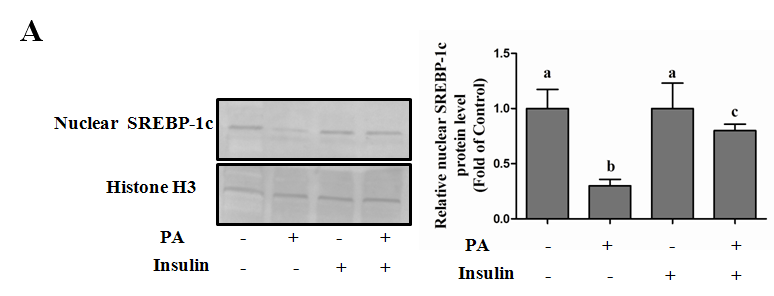


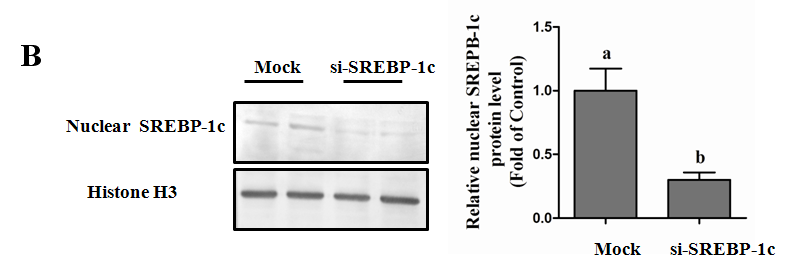


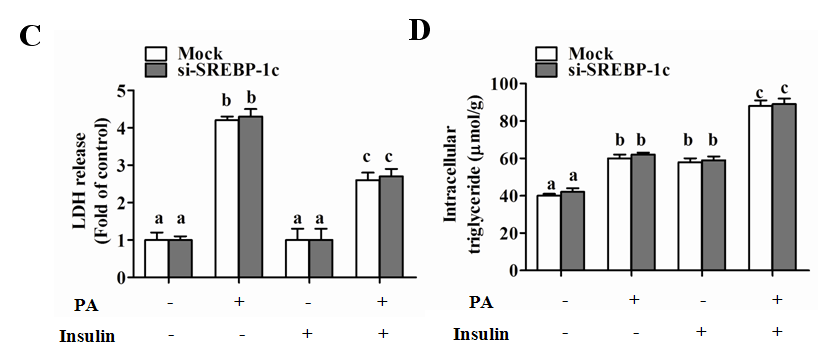


**Figure S6.** Silencing SREBP-1c did not block the protective role of insulin against palmitate-induced cell death. HepG2 cells were exposed to palmitic acid (PA, 400 µM) with insulin pretreatment for 1 h. Cells were transfected with either scrambled or SREBP-1c siRNA. After transfected with either scrambled or SREBP-1c siRNA, cells were treated with palmitic acid (PA, 400 µM) with insulin (200 nM) pretreatment for 1 h. (**A**) Immunoblotting assay for nuclear SREBP-1c; (**B**) Silencing efficiency for SREBP-1c was detected by immunoblotting assay; (**C**) LDH in the cultured medium was detected as described in the Methods; (**D**) Intracellular triglyceride contents were detected as described in the Materials and methods. Each test was performed at least 3 times. Bars with different characters differ significantly (*p* < 0.05).


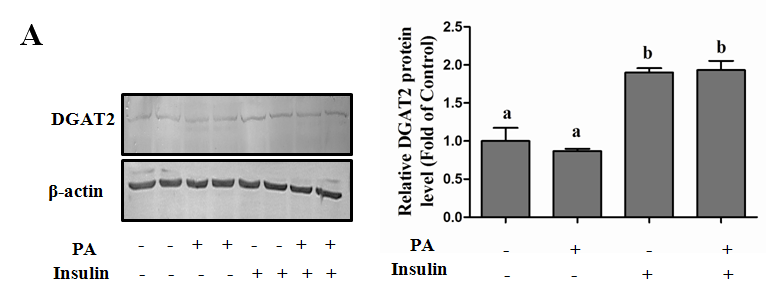


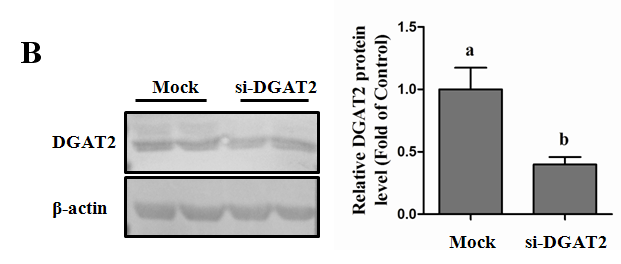


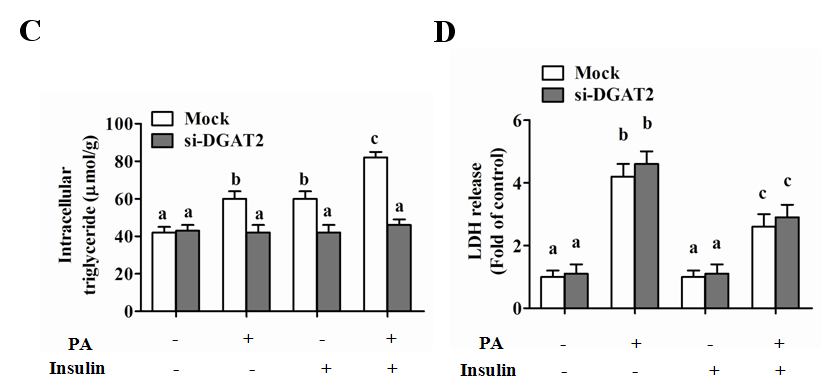


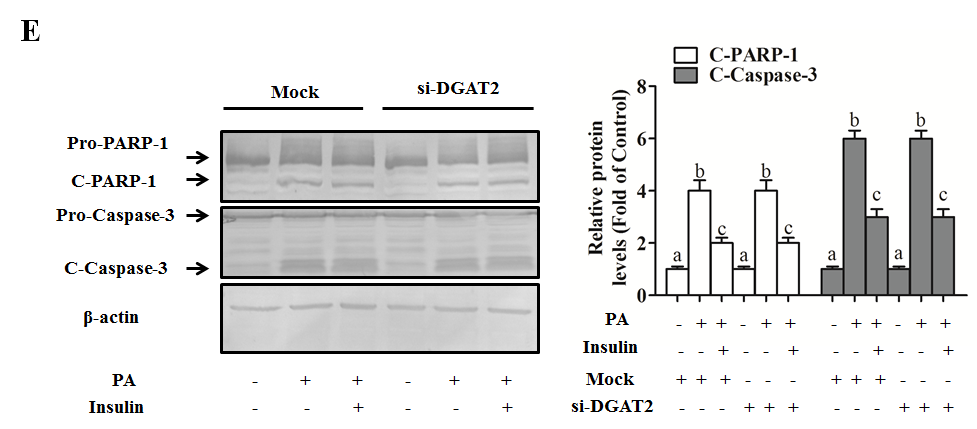


**Figure S7.** Knocking-down DGAT-2 did not inhibite insulin protected lipotoxicity induced by palmitate. HepG2 cells were exposed to palmitic acid (PA, 400 µM) with insulin (200 nM) pretreatment for 1 h. Cells were transfected with either scrambled or DGAT2 siRNA. After transfected with either scrambled or DGAT2 siRNA, cells were treated with palmitic acid (PA, 400 µM) with insulin (200 nM) pretreatment for 1 h. (**A**) Immunoblotting assay for DGAT-2; (**B**) Silencing efficiency for DGAT2 was detected by immunoblotting assay; (**C**) Intracellular triglyceride contents were detected as described in the Materials and methods; (**D**) LDH in the cultured medium was detected as described in the Methods; (**E**) Immunoblotting assay for Caspase-3 and PARP-1. Each test was performed at least 3 times. Bars with different characters differ significantly (*p* < 0.05).
